# Improvement of the Low-Energy Adaptive Clustering Hierarchy Protocol in Wireless Sensor Networks Using Mean Field Games

**DOI:** 10.3390/s24216952

**Published:** 2024-10-30

**Authors:** Unalido Ntabeni, Bokamoso Basutli, Hirley Alves, Joseph Chuma

**Affiliations:** 1 Department of Electrical, Computer and Telecommunication Engineering, Faculty of Engineering, Botswana International University of Science and Technology, Palapye 10071, Botswana; basutlib@biust.ac.bw (B.B.); chumaj@biust.ac.bw (J.C.); 2 Faculty of Information Technology and Electrical Engineering, University of Oulu, 90570 Oulu, Finland; hirley.alves@oulu.fi

**Keywords:** low-energy adaptive clustering hierarchy, wireless sensor network, scalability, mean field games, energy efficiency

## Abstract

The Low-Energy Adaptive Clustering Hierarchy (LEACH) protocol is a widely used method for managing energy consumption in Wireless Sensor Networks (WSNs). However, it has limitations that affect network longevity and performance. This paper presents an improved version of the LEACH protocol, termed MFG-LEACH, which incorporates the Mean Field Game (MFG) theory to optimize energy efficiency and network lifetime. The proposed MFG-LEACH protocol addresses the imbalances in energy consumption by modeling the interactions among nodes as a game, where each node optimizes its transmission energy based on the collective state of the network. We conducted extensive simulations to compare MFG-LEACH with Enhanced Zonal Stable Election Protocol (EZ-SEP), Energy-Aware Multi-Hop Routing (EAMR), and Balanced Residual Energy routing (BRE) protocols. The results demonstrate that MFG-LEACH significantly reduces energy consumption and increases the number of packets received across different node densities, thereby validating the effectiveness of our approach.

## 1. Introduction

Wireless Sensor Networks (WSNs) are a highly significant technological development, enabling the efficient gathering and sharing of data across multiple domains [1]. These networks are composed of small sensor nodes that possess capabilities for sensing, data processing, and wireless communication. WSNs are applied in a variety of areas, including environmental monitoring, healthcare, industrial automation, and smart cities [2,3,4,5,6], etc.

Despite their numerous benefits, WSNs face significant challenges, particularly in terms of energy efficiency and network longevity. Traditional clustering protocols, such as the Low-Energy Adaptive Clustering Hierarchy (LEACH), have been widely used to address these issues. However, LEACH and similar protocols often fail to optimize energy consumption [7,8,9,10] effectively in large-scale networks due to their static and non-adaptive nature resulting in the uneven energy distribution among sensor nodes, leading to premature energy depletion and reduced network lifespan [11,12]. In large-scale deployments, this issue is exacerbated, where the distance between cluster heads (CHs) and the sink node can vary significantly, resulting in increased energy consumption for some nodes.

The motivation for our proposed work is rooted in reducing energy consumption to enhance the durability and efficiency of WSNs. This research aims to contribute to ongoing efforts to create energy-efficient solutions that improve the performance and sustainability of WSNs. The development of such protocols will make WSNs more reliable and cost-effective for a variety of applications, supporting their continued expansion and utility in numerous industries.

### 1.1. Related Works

Several studies have addressed the limitations of the LEACH protocol, as highlighted in [13,14,15,16,17,18,19,20,21,22] and have been summarised in Table 1. One of the challenges with LEACH is the uneven distribution of nodes among different CHs, resulting in varying network performance. To mitigate this issue, MaximuM-LEACH [16] has been introduced, aiming to achieve a more balanced load by equalizing the number of nodes within each cluster. This strategy is designed to prolong the overall network lifespan. In [17], a modified protocol named LEACH-Centralized Modified (LEACH-CM) is introduced to improve upon the existing centralized LEACH (LEACH-C). LEACH-CM primarily involves evaluating the distance between a selected CH and a member node, as well as the distance between the member node and the BS for data transmission. Addressing inter-cluster communication issues in LEACH, ref.  [19] proposes an enhanced energy-saving strategy using inter-cluster ring routing. This approach abstracts all sensor nodes into a ring system model during network topology formation and implements a ring transmission strategy during data transmission. Additionally, ref. [20] introduces improved computation algorithms for LEACH clustering and enhanced information gathering by CHs, enhancing the rate of combined information flow. Moreover, ref. [14] proposes an enhanced version of LEACH, referred to as enhanced LEACH (E-LEACH), to extend network lifetime and improve energy utilization. Scalability concerns of traditional LEACH are addressed in [21] through the introduction of V-LEACH [15]. Furthermore, ref. [13] presents Energy-LEACH and Multihop-LEACH variations to enhance scalability, while ref. [22] focuses on implementing multihop flat-based and cluster-based protocols to improve scalability. Lastly, ref. [18] introduces the Energy-Efficient Scalable Routing Algorithm (EESRA) as an adaptation of LEACH.

In the subject of handling scalability and high node densities in WSNs, one interesting solution is Mean Field Game (MFG), a mathematical framework employed to model decision-making within extensive populations of interacting agents [23,24]. This concept is particularly important in the field of economics [25], network security [26,27,28], and traffic networks [29,30] and has also been applied to wireless networks [31,32,33,34,35].

**Table 1:** Comparison of LEACH protocol variants.

Algorithm	References	Year	Key Features
LEACH	[36,37]	2000	Uneven distribution of nodes among CHs, rotating CHs, localized data aggregation and limited network lifespan.
Energy-LEACH	[13]	2009	Considers the residual energy of nodes as the primary metric for CH selection.
Multihop-LEACH	[13]	2009	Employs multi-hop communication and selects optimal paths to reduce energy consumption and prolong the network lifetime.
E-LEACH	[14]	2013	Introduces enhancements in cluster formation and CH selection processes, extension of network lifetime and improved energy utilization.
V-LEACH	[15]	2013	Improves the lifetime of the network by introducing a vice-CH to mitigate the impact of CH failures.
MaximuM-LEACH	[16]	2018	Balanced load distribution among clusters and prolonged network lifespan.
LEACH-C	[17]	2021	Centralized approach, considers distance for CH selection and data transmission and considers active node count for CH determination.
ESSRA	[18]	2022	Sink node makes decisions about CH selection to reduce energy consumption and it considers residual energy levels and distance from CHs.
MFG-LEACH	This work	2024	Incorporates the Mean Field Games framework into the data transmission phase of LEACH to select the optimal energy for transmitting.

In [31], the focus is on minimizing transmission costs in an IoT framework with a hybrid access point (HAP) and multiple sensor nodes, employing a Stackelberg game extended to a dynamic game for efficient power control. Power control for energy efficiency in a wireless network is addressed in [32] with numerous transmitters, modeling it as a dynamic game based on available energy and channel conditions. Ref. [33] examines uplink power control using the non-orthogonal multiple access (NOMA) protocol in scenarios with many users, establishing an equilibrium strategy via MFG theory. Power management in densely populated small-scale cellular networks is investigated in [34], integrating MFG theory to ensure unique mean field equilibrium and network stability. Finally, ref. [35] focuses on optimizing quality of service (QoS) in cellular networks through decentralized optimization using nonlinear MFG control theory.

### 1.2. Contribution

Within the MFG framework, the solutions in [31,32,33,34,35] focus on various wireless networking applications. However, despite this extensive research, there is a notable gap in the literature regarding the application of MFG theory to the routing processes in WSNs. The main contributions of the work can be outlined as follows:

1. The study focuses on enhancing the data transmission phase of the LEACH protocol by introducing the application of the MFG framework, which models the interactions among sensor nodes as a game. Each node independently optimizes its transmission energy based on the collective state of the network.
2. This work introduces an MFG model that considers two network dynamics; distance and the remaining energy of the nodes. This model holds significance as the incorporation of these parameters is crucial for enhancing the performance of the LEACH protocol.
3. Scalability analysis is performed in this work as the proposed technique is evaluated across different node densities.

### 1.3. Paper Outline

The paper follows an organized format. The introduction is presented in Section 1, covering the motivation and contribution of the work. Section 2 describes the system model used in the study, formulates the problem and then introduces the differential game model and optimal control framework, respectively, which are the building blocks of MFG. The notations used in this section and the subsequent ones are provided in Table A1. Section 3 gives an overview of the MFG framework and Section 4 introduces the MFG-LEACH protocol and the solution analysis. Section 5 presents the numerical analysis with a specific emphasis on the comparison between MFG-LEACH and other routing protocols. Section 6 provides a discussion of the findings. Finally, Section 7 summarizes the main findings, the contributions of the paper, and provides closing remarks on the study’s significance along with suggestions for future work.

## 2. System Model

### 2.1. Network Model

In this study, we examine a WSN consisting of *K* individual sensor nodes, which are randomly deployed. Each sensor node in this network is equipped with components enabling sensing, computational processing, and wireless communication functionalities. The network architecture includes a sink node located at the edge of the deployment area. Data transmission within this WSN is governed by the LEACH protocol which facilitates efficient data transmission through dynamic clustering and rotating CHs. The cluster’s channel model is presumed to adhere to a free space propagation model. Additionally, the channels between CHs and the sink node are multi-path fading channels. The sink plays a role in aggregating data collected from the dispersed sensor nodes.

### 2.2. LEACH Protocol

**Figure 1:**
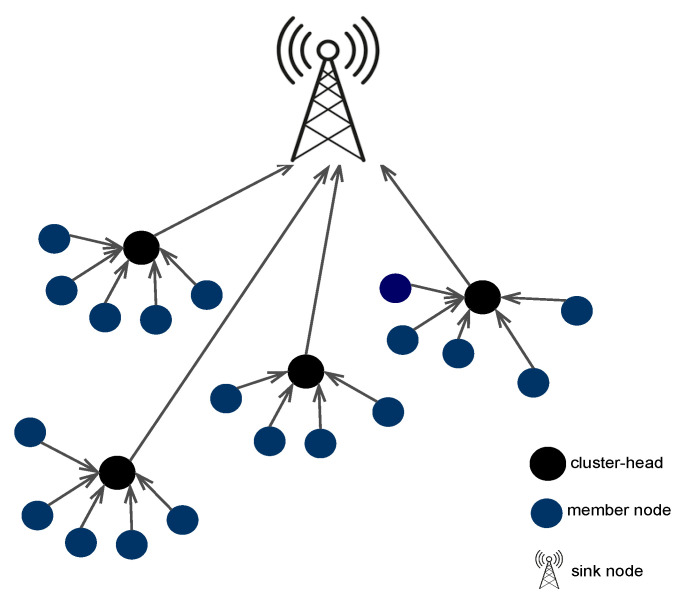
Network model [38].

The LEACH protocol is adopted by the nodes in which nodes self-organize into clusters as shown in Figure 1. LEACH operates in two phases: the *setup phase* and the *steady state phase*.

#### 2.2.1. Setup Phase

The selection of CHs follows a probabilistic model, aiming to allocate energy usage evenly throughout the network according to the mathematical formulation [39]:

(1) $$P\left(\mathrm{CH}\right)=\frac{1}{1-P\cdot (r\cdot \mathrm{mod}\cdot \frac{1}{P})}$$

where P(CH) denotes the probability threshold dictating whether a specific node will act as a CH in the ongoing round, while *P* signifies the percentage of nodes designated to become CHs—a parameter representing the optimal proportion of CHs at any point. The variable *r* denotes the current round number, and the operation mod refers to the modulus operation. CHs are responsible for collecting data from member nodes. To balance energy consumption, CHs rotate periodically.

#### 2.2.2. Steady State Phase

Nodes transmit data through a single hop to a CH, which aggregates the data and forwards them to the sink. The energy expended by node *k* in transmitting *m*-sized bits of data is [40]

(2) $$E_{k}=\left\{\begin{array}{cc} E_{\mathrm{elec}}\cdot m+E_{\mathrm{fs}}\cdot m\cdot d^{2} & \mathrm{if}\;d<d_{\mathrm{th}}\\ E_{\mathrm{elec}}\cdot m+E_{\mathrm{amp}}\cdot m\cdot d^{4}, & \mathrm{if}\;d>d_{\mathrm{th}}, \end{array}\right.$$

$E_{\mathrm{elec}}$ denotes the node electronics energy, and $E_{\mathrm{amp}}$ pertains to the energy required for amplification of a signal in a multi-path model. The variables *d* and $d_{\mathrm{th}}$ denote the distance from the transmitter (node or CH) to the receiver (CH or sink), and a predefined threshold distance, respectively. $E_{\mathrm{fs}}$ represents the energy required for amplification of a signal in open space and is utilized when the distance from the source to the destination is below $d_{\mathrm{th}}$. The energy expended in receiving *l* bits of data is [40]

(3) $$E_{R}=\left(E_{\mathrm{elec}}+E_{\mathrm{DA}}\right)\cdot l$$

where $E_{\mathrm{DA}}$ refers to the energy expenditure necessary for aggregating each bit of data.

#### 2.2.3. Complexity Analysis

LEACH operates by probabilistically selecting CHs among nodes, where each node computes a threshold based on a random number and a probability threshold. The complexity is characterized by how this process scales as the network grows. Each node independently calculates a threshold based on a random number and a probability value. This operation is local to each node and involves basic arithmetic operations, typically resulting in a constant time complexity for each node, denoted as O(1). Nodes are organized into clusters based on the thresholds they calculate. This clustering process involves nodes communicating and potentially establishing CHs. In a network with *K* nodes, each node typically interacts with a constant number of neighbors (or potential CHs), resulting in a constant overhead per node, often denoted as O(1). Since the key operations—threshold calculation and cluster formation— are repeated for each node in the network, the overall complexity of LEACH in terms of node count *K* is O(K). This linear scalability means that as the number of nodes increases, the computational and communication load increases proportionally.

### 2.3. Problem Formulation

#### 2.3.1. Scenario

As shown in (2), the energy required to transmit data increases with distance due to path loss. For shorter distances, the energy consumption is modeled with a free space propagation model, which considers path loss to be proportional to $d^{2}$. For longer distances, the multipath fading model is used, where the path loss is proportional to $d^{4}$.

The energy formulation used in the LEACH protocol has certain limitations and downsides. The model uses a fixed threshold distance $\left(d_{\mathrm{th}}\right)$ to switch between the $d^{2}$ and $d^{4}$ propagation models. This creates a sharp transition, which may not accurately reflect the gradual change in path loss and signal-to-interference plus noise ratio (γ) experienced in real environments. The fixed threshold does not account for varying environmental conditions, such as obstacles, terrain, and interference. Additionally, the model leads to an overestimation or underestimation of the energy required for transmission, especially for distances close to $d_{\mathrm{th}}$. Nodes located just above or below the threshold may experience significantly different energy consumption rates, leading to unbalanced energy depletion across the network. This can cause some nodes to die prematurely, reducing coverage and effectiveness.

Path loss serves as an indicator of communication reliability due to its direct impact on γ. The path loss *(PL)* can be expressed as [41,42]:

(4) $$PL\left(dB\right)=PL\left(d_{0}\right)+10n\log\left(\frac{d}{d_{0}}\right)$$

or

(5) $$PL\left(dB\right)=P_{t}-P_{r}$$

where $d_{0}$ is the reference point in 1 km, $P_{t}$ is the transmit power, $P_{r}$ is the receive power and *n* is the path loss exponent. Higher path loss results in weaker received signal strength and lower γ, which indicates poor signal quality. γ is defined as

(6) $$\gamma =\frac{P_{r}}{P_{\mathrm{int}}+P_{\mathrm{noise}}}$$

where $P_{\mathrm{int}}$ is the total power of all interfering signals and $P_{\mathrm{noise}}$ is the power of the background noise present in the system, which includes thermal noise and other sources of noise.

#### 2.3.2. Optimization Problem

In this scenario, each battery-constrained node *k* seeks to minimize its transmission energy $E_{k}$

(7) $$E_{k}=P_{k}\cdot t$$

where $P_{k}$ is the transmission power of node *k*, and $P_{k}$ = $P_{t}$. In our work, network dynamics of remaining battery energy ($E^{r}$) and γ will be considered during the data transmission phase. The objective of each node is to minimize energy consumption while ensuring that γ remains above a set threshold $\left(\gamma_{\mathrm{th}}\right)$. Thus, the optimization problem at each node is then formulated as

(8) $$\begin{array}{cc} \mathrm{Minimize}: & \;E_{k}\\ \mathrm{subject}\mathrm{to}: & \;\gamma_{k}\geq \gamma_{\mathrm{th}}\\ \; & \;E_{\min}\leq E_{k}\leq E_{\max} \end{array}$$

where $E_{\min}$ and $E_{\max}$ puts both lower and upper bounds on $E_{k}$, $E_{\max}\leq E^{r}$ and the value of $\gamma_{\mathrm{th}}$ is constant for all the nodes.

Since the nodes share the transmission medium, the transmission power and channel conditions of one node can affect γ and energy consumption of other nodes due to interference. This creates a conflict of interest, where the actions of one node can have negative externalities on others. Thus, optimizing each node independently, without accounting for interactions and dependencies among them, may not result in the most efficient utilization of energy from a global network perspective and could lead to sub-optimal overall performance.

Game theory emerges as a viable optimization technique capable of considering node interactions [43,44], offering a distributed solution to the optimization problem. In this context, node strategies are adjusted in response to evolving network conditions and the strategies of other nodes.

### 2.4. Differential Game Model

The scenario is modeled as a differential game [45], wherein nodes make continuous, time-varying decisions, and the outcome is influenced not only by node strategies but also by the dynamic system.

#### 2.4.1. Framework

The differential game (G) framework comprises five sets: G={K,E,S,V,C}. In this setting, the group of players K={1,…,K} denotes deployed nodes, with each node functioning as a rational decision maker. The action space $E=[E_{\min},E_{\max}]$ defines potential transmit energy levels. The state space S is characterized by γ and $E_{k}^{r}$, where the state of a node is denoted as $s_{k}\in S$ and $s_{k}\left(t\right)=\left[E_{k}^{r}\left(t\right),\gamma_{k}\left(t\right)\right]$. Both states’ evolution is presumed to adhere to the Ornstein–Uhlenbeck dynamics, a mathematical framework offering insight into real-world dynamic phenomena [46]. The cost function $c_{k}\left(t\right)\in C$ for a node at any given time considers both receiver performance and transmit energy, and is defined as [47]

(9) $$c_{k}\left(t\right)={\left(\gamma_{k}\left(t\right)-\gamma_{\mathrm{th}}\left(t\right)\right)}^{2}+\mu E_{k}\left(t\right),$$

where the term ${\left(\gamma_{k}-\gamma_{\mathrm{th}}\right)}^{2}$ signifies the performance-related term, and $\mu E_{k}\left(t\right)$ denotes the transmit energy-related term. μ is a weighting factor that balances the importance of receiver performance against energy consumption.

To determine the value of $E_{k}$ that will minimize $c_{k}\left(t\right)$ in this dynamic system, each node formulates an optimal control policy $v_{k}^{*}\in V$ [48] defined as

(10) $$\begin{array}{cc} v_{k}^{*}\left(0\to T\right)=\arg\;\min_{E_{k}\left(0\to T\right)}\; & E\left[\int_{0}^{T}c_{k}\left(t\right)dt+c_{k}\left(T\right)\right],\forall k\\ \mathrm{subject}\mathrm{to}: & s_{k}\left(t\right)=\left\{E_{k}^{r}\left(t\right),\gamma_{k}\left(t\right)\right\},\forall k \end{array}$$

where t∈[0,T] and $c_{k}\left(T\right)$ represents the final condition which characterizes the system’s cost at final time *T*.

#### 2.4.2. Optimal Control

The optimal control for each node is derived by solving the Hamilton–Jacobi–Bellman (HJB) equation, which provides the value function $\left(u_{k}\right)$ representing the minimum cost-to-go from any given state [48]. The HJB is expressed as in (11) and as the Hamiltonian in (12) [34]:

(11) $$-\delta_{t}u_{k}\left(t,s_{k}\left(t\right)\right)=\min_{E_{k}\left(t\right)}\;\left[c_{k}\left(t,s_{k}\left(t\right),E_{k}\left(t\right)\right)+\nabla u_{k}\left(t,s_{k}\left(t\right)\right)\cdot \delta_{t}s_{k}\left(t\right)\right]$$

where the term $-\delta_{t}u_{k}\left(t,s_{k}\left(t\right)\right)$ indicates how the value function changes with a small increment in time, $c_{k}\left(t,s_{k}\left(t\right),E_{k}\left(t\right)\right)$ is the intermediate cost which quantifies the instantaneous cost incurred due to the chosen action in the given state at that time, $\nabla u_{k}\left(t,s_{k}\left(t\right)\right)$ represents the value function’s gradient with respect to $s_{k}\left(t\right)$, which points in the direction of the steepest increase in the value function and $\delta_{t}s_{k}\left(t\right)$ denotes the change in $s_{k}\left(t\right)$ with respect to *t* and it captures how the state of the system evolves over time. To find $E_{k}$, it is necessary to solve *K* HJB equations concurrently. In densely populated WSNs, this approach becomes impractical because the number of simultaneous partial differential equations (PDEs) increases with the number of nodes. As a result, the complexity of optimizing energy consumption and maintaining network performance grows proportionally with the network. To address this challenge, we leverage the MFG framework, which offers a robust method for analyzing and optimizing large-scale systems.

(12) $$H(s_{k}\left(t\right),E_{k}\left(t\right),\nabla u_{k}\left(t,s_{k}\left(t\right)\right)=\min_{E_{k}\left(t\right)}\left[c_{k}\left(s_{k}\left(t\right),E_{k}\left(t\right)\right)+u_{k}\left(t,s_{k}\left(t\right)\right)\cdot \delta_{t}s_{k}\left(t\right)\right]$$

## 3. Mean Field Game (MFG) Framework

MFG is a framework designed to address scenarios in which a vast number of rational agents (nodes), interact with one another. The key assumptions underlying this framework are as follows:

1. Nodes are rational and are motivated to optimize their individual objectives.
2. As the number of nodes tends to infinity, a mean field approximation is applied.
3. Nodes can only observe and react to the aggregate behavior of the population.
4. This aggregate behavior can be described through a distribution referred to as the mean field.
5. The actions taken by nodes have an impact on the system’s overall dynamics.

In MFG, at a specific moment in time, the mean field corresponds to the probability distribution describing the states across the entire group of nodes. It is represented by a mean field variable (m(t,s)) which is expressed as [49]

(13) $$m\left(t,s\right)=\lim_{K\to \infty }\frac{1}{K}\sum_{i=1}^{K}I_{\left[s_{k}\left(t\right)=s\right]}$$

where $\lim_{K\to \infty }$ indicates that the operation is conducted in the limit as *K* approaches infinity. The term $\frac{1}{K}\sum_{i=1}^{K}I_{\left[s_{k}\left(t\right)=s\right]}$ represents an average over *K* nodes, where $I_{\left[s_{k}\left(t\right)=s\right]}$ is an indicator function that takes the value 1 if $s_{k}\left(t\right)=s$.

The MFG framework is described by a system of two interconnected PDEs, namely the HJB Equation (11) and the Fokker–Planck–Kolmogorov (FPK) equation, denoted by

(14) $$\delta_{t}m\left(t,s_{k}\right)+\nabla \left(m\left(t,s_{k}\right)\cdot \delta_{t}s_{k}\left(t\right)\right)=0$$

where $\delta_{t}m\left(t,s_{k}\right)$ represents how the variable $m(t,s_{k})$ changes over time and $\delta_{t}s_{k}\left(t\right)$ represents the rate of change of $s_{k}$ with respect to *t*. The equation describes how the probability density of a system’s state evolves over time.

**Figure 2:**
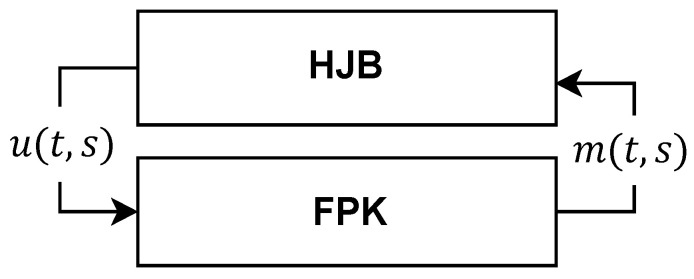
MFG operation [47].

The HJB equation describes the value function of each node which represents the expected cost that it seeks to minimize [48]. The FPK equation characterizes the evolution of the distribution of the system’s state over time. The mean field establishes the relationship between the value functions and the distribution of the states, connecting the HJB equation and the FPK Equation [50]. Thus, instead of solving *K* HJB equations, the strategy of a node, $E_{k}$, is influenced by the collective state of all the other nodes. The interaction of these two equations is depicted in Figure 2.

### Complexity Analysis

In this work, the mean field represents a statistical distribution characterizing the state of a two-dimensional system at a specific time. It quantifies the likelihood of various states across the player set, as indicated by (13). The computational challenge lies in determining this mean field, which entails assessing the probability of *S* states across *K* players at any given time, t∈[0,T]. The summation in the equation encompasses all players, indexed by *i*, with *K* representing the number of players. Hence, the computational complexity scales proportionally with the player count. Additionally, for each time point *t*, the mean field computation is necessary, leading to a scaling complexity with the number of time instances, *T*. Moreover, as the mean field is computed for each achievable state *s* within the set S, the total possible states, *S*, further contribute to the complexity. Taking these factors into consideration, the computational complexity of mean field determination, integrated into LEACH to formulate MFG-LEACH, can be represented as O(K·T·S). This complexity arises from the necessity to iterate through all players, time instances, and states.

## 4. MFG-LEACH Protocol

The routing mechanism of the MFG-LEACH protocol is an enhancement of the traditional LEACH protocol. The primary objective is to balance energy consumption among sensor nodes by considering the collective state of the network. Similar to traditional LEACH, MFG-LEACH operates in rounds and begins with the setup phase, where clusters are formed. Each sensor node independently decides whether to become a CH based on a probabilistic approach that considers a set threshold. Once a CH is selected, the steady-state phase begins where data transmission occurs. Unlike traditional LEACH, which uses a static approach to compute $E_{k}$ according to (2), in MFG-LEACH, to derive $E_{k}$, we utilize a coupled system of two equations: the HJB and FPK equations. The FPK equation evolves forward in time and dictates the evolution of the mean field. Conversely, the HJB equation evolves backward in time and governs the computation of the optimal control policy for node *k*. The continuous feedback loop is key to ensuring that the system dynamically adapts to changes, such as varying node densities, energy levels, or network conditions. As these environmental factors change, the mean field shifts, prompting agents to modify their strategies dynamically.

### 4.1. LEACH vs. MFG-LEACH

The complexity of MFG-LEACH is significantly higher than that of LEACH. By comparing the complexities of LEACH and MFG-LEACH (Table 2), it is evident that MFG-LEACH is much more complex than LEACH. This is because LEACH scales linearly with the number of nodes while MFG-LEACH introduces complexity due to the additional parameters *T* and *S*. The increased complexity of MFG-LEACH due to its integration of MFG aims for more optimized energy consumption but at the cost of higher computational demands.

### 4.2. Solution Analysis

The solution of the coupled PDEs (HJB and FPK) in MFG-LEACH is complex and often requires numerical methods particularly finite difference methods (FDM) for practical computation [51,52]. FDM discretizes both the spatial and temporal dimensions of the equations and approximates derivatives using finite difference techniques. The resulting system of algebraic equations is then solved iteratively.

To utilize this method effectively, the continuous variables, time, energy and γ are discretized into a grid with dimensions X×Y×Z. The decision-making for the optimal control policy takes place within this discretized space and the variables are used to approximate the solutions of the PDEs which will evolve in the three-dimensional space $\left(0,T\right)\times \left(E_{\min},E_{\max}\right)\times \left(0,\gamma_{\mathrm{th}}\right)$ with steps in time ($\delta_{t}$), energy ($\delta_{E}$), and signal-to-interference plus noise ratio ($\delta_{\gamma }$) defined as follows [51]:

(15) $$\delta_{t}=\frac{T}{X}$$

(16) $$\delta_{E}=\frac{E_{\max}-E_{\min}}{Y}$$

(17) $$\delta_{\gamma }=\frac{\gamma_{\mathrm{th}}}{Z}$$

For the FPK Equation (18), the Lax–Friedrichs scheme is applied to obtain (19).

(18) $$\delta_{t}m\left(t,s\right)+\nabla_{E}m\left(t,s\right)E\left(t\right)+\nabla_{\gamma }m\left(t,s\right)\gamma \left(t\right)=0$$

(19) $$\begin{array}{cc} M(i+1,j,l) & =\frac{1}{2}[M\left(i,j-1,l\right)+M\left(i,j+1,l\right)\\ + & \;M(i,j,l-1)+M(i,j,l)]\\ + & \;\frac{1}{2\delta_{E}}[M\left(i,j+1,l\right)E\left(i,j+1,l\right)\\ - & \;M(i,j-1,l)E(i,j-1,l)]\\ + & \;\frac{1}{2\delta_{\gamma }}[M\left(i,j,l-1\right)E\left(i,j,l-1\right)\gamma \left(i,j,l-1\right)\\ - & \;M(i,j-1,l+1)E(i,j,l+1)\gamma (i,j,l+1)] \end{array}$$

where M(i,j,l) denotes the value of m(t,s) at discrete points defined by time instant *i*, energy level *j*, and γ level *l* in the discretized grid. The terms E(i,j,l) and γ(i,j,l) represent the values of transmission energy ($E_{k}$) and γ, respectively. Employing the Lax–Friedrichs scheme allows us to numerically solve the FPK equation and calculate updated values of M(i+1,j,l).

FDM cannot be used to solve the HJB equation due to the complexities introduced by the Hamiltonian. Instead, we reformulate the HJB equation using its corresponding optimal control problem to obtain [47]:

(20) $$\begin{array}{cc} \min_{E_{k}\left(t\right)} & E\left[\int_{0}^{T}c_{k}\left(t\right)dt+c_{k}\left(T\right)\right] \end{array}$$

$$\begin{array}{cc} \mathrm{subject}\mathrm{to}: & \delta_{t}m\left(t,s\right)+\nabla_{E}m\left(t,s\right)E\left(t\right)\\ \; & +\nabla_{\gamma }m\left(t,s\right)\gamma \left(t\right)=0. \end{array}$$

In this formulation, we introduce a Lagrange multiplier λ(t,s) as depicted in (21), assuming $c_{k}\left(T\right)=0$. The incorporation of λ(t,s) is succeeded by discretizing (21), yielding (22) [47].

A joint finite difference algorithm is subsequently developed using the Lax–Friedrichs scheme and Lagrange relaxation. This algorithm is designed to solve the coupled HJB and FPK equations, thereby obtaining the optimal power control policy. Algorithm 1 outlines the steps to update the mean field distribution, Lagrange multipliers (27), and energy levels (26) to achieve the optimal control policy.

(21) $$\begin{array}{cc} L(m(t,s),E(t,s),\lambda (t,s)) & =E\left[\int_{0}^{T}c_{k}\left(t\right)dt+c_{k}\left(T\right)\right]\\ + & \;\int_{0}^{T}\int_{E_{\min}}^{E_{\max}}\int_{0}^{\gamma_{\mathrm{th}}}\lambda \left(t,s\right)(m\left(t,s\right)+\nabla_{E}m\left(t,s\right)E\left(t\right)\\ + & \;\nabla_{\gamma }m\left(t,s\right)\gamma \left(t\right))dtdEd\gamma \end{array}$$

(22) $$\begin{array}{cc} L_{d} & =\delta_{t}\delta_{E}\delta_{\gamma }\sum_{i=1}^{X+1}\sum_{j=1}^{Y+1}\sum_{l=1}^{Z+1}[M\left(i,j,l\right)C\left(i,j,l\right)\\ + & \;\lambda (i,j,l)(\zeta +\rho +\chi )] \end{array}$$

(23) $$\begin{array}{cc} \zeta & =\frac{1}{\delta_{t}}[M\left(i+1,j,l\right)-\frac{1}{2}(M\left(i,j+1,l\right)\\ \; & \;+M(i,j-1,l)+M(i,j,l+1)+M(i,j,l-1))] \end{array}$$

(24) $$\begin{array}{cc} \rho & =\frac{1}{2\delta_{E}}=[M\left(i,j+1,l\right)E\left(i,j+1,l\right)\\ \; & \;-M(i,j-1,l)E(i,j-1,l)] \end{array}$$

(25) $$\begin{array}{cc} \chi & =\frac{1}{2\delta_{\gamma }}[M\left(i,j,l+1\right)E\left(i,j,l+1\right)\gamma \left(i,j,l+1\right)\\ - & \;M(i,j,l-1)E(i,j,l-1)\gamma (i,j,l-1)] \end{array}$$

(26) $$\begin{array}{cc} \frac{\delta L_{d}}{\delta E(i,j,l)} & =\sum_{j=1}^{Y+1}\sum_{l=1}^{Z+1}M\left(i,j,l\right)\frac{\delta C(i,j,l)}{\delta E(i,j,l)}\\ + & \;\left[\frac{M(i,j,l)}{2\delta_{E}}+\frac{M(i,j,l)\gamma (i,j,l)}{2\delta_{\gamma }}\right][\lambda \left(i,j+1,l\right)\\ - & \;\lambda (i,j-1,l)] \end{array}$$

(27) $$\begin{array}{cc} \lambda (i-1,j,k) & =\frac{1}{2}\left[\lambda \left(i,j+1,l\right)+\lambda \left(i,j-1,l\right)\right]\\ + & \frac{1}{2}\left[\lambda \left(i,j,l+1\right)+\lambda \left(i,j,l+1\right)\right]\\ - & \frac{1}{2}\delta_{t}E\left(i,j,l\right)\left[\frac{\gamma (i,j,l)}{\delta_{\gamma }}+\frac{1}{\delta_{\gamma }}\right][\lambda \left(i,j+1,l\right)\\ - & \gamma \left(i,j-1,l\right)]+\delta_{t}C\left(i,j,l\right) \end{array}$$

**Algorithm 1:** Mean Field Equilibrium
1:
**Initialization:**
2:M(0,0,0):= joint mean field distribution3:λ(X+1,0,0):= initial Lagrangian parameters4:E(X+1,0,0):= initial energy levels5:**for** i=1 to *X* **do**6:  **for** j=1 to *Y* **do**7:    **for** k=1 to *Z* **do**8:      **Update mean field:**9:      Using M(i+1,j,l) (19)10:      **Update Lagrangian parameter:**11:      λ(i−1,j,l) (27)12:      **Update energy levels:**13:      E(i−1,j,l) (26)14:    **end for**15:  **end for**16:
**end for**



To achieve optimality, the decision variables ($E^{*}$ and $M^{*}$) must satisfy the Karush–Kuhn–Tucker (KKT) conditions. Using Algorithm 1, $M^{*}$ is first calculated, and then $E^{*}$ ($E_{k}$) is determined according to (26). This process allows the MFG-LEACH protocol to compute the optimal transmission energy $E_{k}$.

## 5. Numerical Analysis

### 5.1. Simulation Setup

In this section, we provide an assessment of the proposed scheme, comparing it with other established routing protocols including EZ-SEP [53], EAMR [54], BRE [55]. The parameters used in the simulation are presented in Table 3.

### 5.2. Node Density

The node density is varied from sparse to dense (0.001, 0.01, 0.05 and 0.1 nodes per square meter (m^2^)), allowing for the assessment of the scalability of MFG-LEACH. The nodes were distributed randomly.

### 5.3. Results

We computed $E_{k}$ using the MFG-LEACH protocol. To demonstrate the performance of the proposed approach, we examined energy consumption and throughput across a range of node densities, from sparse to dense.

## 6. Discussion

### 6.1. Energy Consumption Per Round

**Figure 3:**
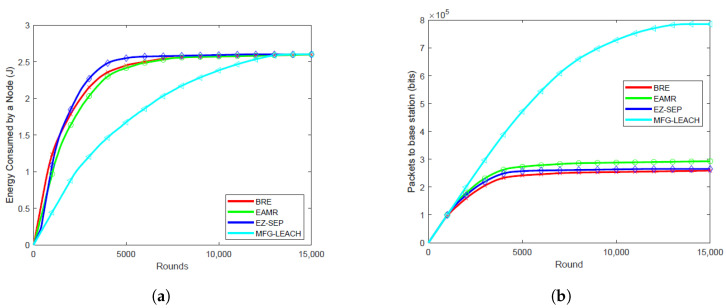
Performance comparison of energy consumption and throughput at 0.01 node density of BRE, EAMR, EZ-SEP, and MFG-LEACH protocols. (**a**) Comparison of energy consumption at 0.01 node density between BRE, EAMR, EZ-SEP, and MFG-LEACH. (**b**) Comparison of throughput at 0.01 node density between BRE, EAMR, EZ-SEP, and MFG-LEACH.

**Figure 4:**
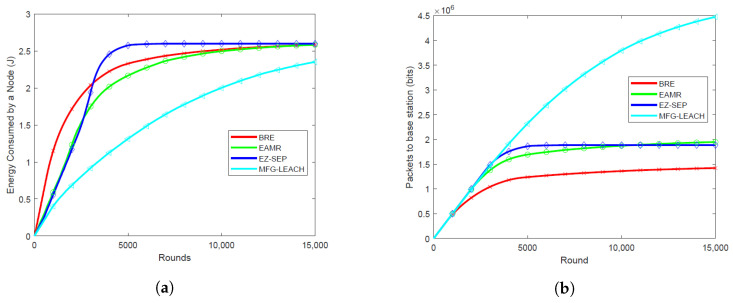
Performance comparison of energy consumption and throughput at 0.05 node density of BRE, EAMR, EZ-SEP, and MFG-LEACH protocols. (**a**) Comparison of energy consumption at 0.05 node density between BRE, EAMR, EZ-SEP, and MFG-LEACH. (**b**) Comparison of throughput at 0.05 node density between BRE, EAMR, EZ-SEP, and MFG-LEACH.

**Figure 5:**
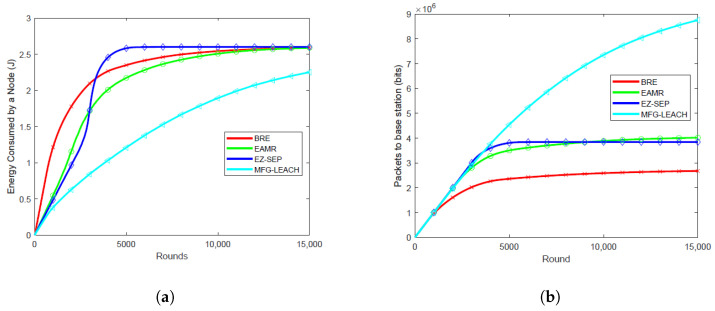
Performance comparison of energy consumption and throughput at 0.1 node density of BRE, EAMR, EZ-SEP, and MFG-LEACH protocols. (**a**) Comparison of energy consumption at 0.1 node density between BRE, EAMR, EZ-SEP, and MFG-LEACH. (**b**) Comparison of throughput at 0.1 node density between BRE, EAMR, EZ-SEP, and MFG-LEACH.

**Figure 6:**
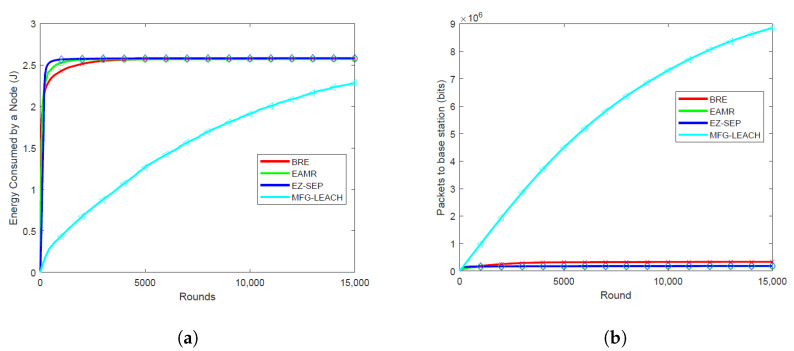
Performance comparison of energy consumption and throughput at 0.001 node density of BRE, EAMR, EZ-SEP, and MFG-LEACH protocols. (**a**) Comparison of energy consumption at 0.001 node density between BRE, EAMR, EZ-SEP, and MFG-LEACH. (**b**) Comparison of throughput at 0.001 node density between BRE, EAMR, EZ-SEP, and MFG-LEACH.

Energy consumption per round is critical as it directly impacts the overall network lifetime. The energy consumption of MFG-LEACH is notably lower compared to EZ-SEP, EAMR and BRE across all node densities, as illustrated in Figure 3a, Figure 4a, Figure 5a and Figure 6a. For all the node densities, 0.001, 0.01, 0.05 and 0.1, the plots show that the other protocols consumed more energy per round than MFG-LEACH. For instance, after 1000 rounds at a node density of 0.1, EAMR, EZ-SEP and BRE have consumed 0.547 J, 0.487 J and 1.22 J, respectively, while node energy consumption under MFG-LEACH routing is 0.375J. The other protocols consume more energy per round because they do not have MFG-LEACH’s adaptive control policy, which dynamically adjusts transmission energy in response to changes in network dynamics.

### 6.2. Throughput

Throughput helps in visualizing the correlation between the number of packets received by the base station in each round across various node densities. Higher throughput is advantageous as it ensures that more data packets are successfully transmitted to the base station, enhancing data collection efficiency and overall network reliability. Figure 3b, Figure 4b, Figure 5b and Figure 6b illustrate the packet reception by the base station at node densities of 0.01, 0.05, 0.1, and 0.001, respectively. The results show that MFG-LEACH outperforms EZ-SEP, EAMR and BRE in terms of throughput across all examined node densities. The adaptive nature of MFG-LEACH ensures that nodes remain active longer and do not die prematurely. As a result, more data packets are transmitted to the base station before the nodes deplete their energy.

### 6.3. Network Lifetime

**Figure 7:**
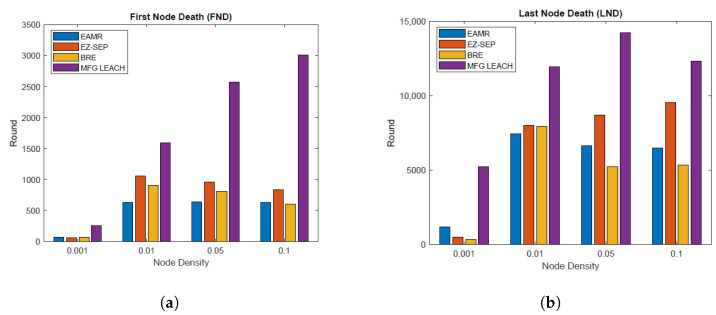
Network lifetime analysis based on the first and last node death events using the MFG-LEACH protocol. (**a**) Time until the first node death. (**b**) Time until the last node death.

The purpose of this metric is to evaluate how long the network remains operational. The network lifetime is evaluated through two key metrics: first node death (FND), representing the round when the first node depletes its energy, and LND, indicating the round when the last active node becomes inactive. These metrics are illustrated in Figure 7a,b. The proposed approach enhances network lifetime, as evidenced by the prolonged activity of nodes across all node densities compared to EZ-SEP, EAMR and BRE. This delay in FND and LND is attributed to the lower energy consumption observed in the protocol, resulting in longer node activity compared to other approaches. Extending FND and LND means that the network remains operational for a longer period, which is crucial for applications requiring sustained sensor activity and data collection.

### 6.4. Scalability

Scalability was assessed by testing MFG-LEACH across node densities ranging from 0.001 to 0.1 nodes per m^2^. MFG-LEACH consistently maintained high performance levels, demonstrating its robustness in both sparse and dense network conditions compared to EZ-SEP, EAMR and BRE. This characteristic of MFG-LEACH’s ability to handle large populations indicates its adaptability and flexibility.

## 7. Conclusions

In our study, we introduced MFG-LEACH, an enhanced LEACH protocol leveraging MFG theory to tackle energy optimization, scalability, and network longevity challenges in WSNs. Our approach enables dynamic adjustment of transmission strategies, resulting in balanced energy consumption and improved scalability and throughput compared to traditional protocols such as EAMR, EZ-SEP, and BRE, as demonstrated in Table 4. Additionally, the optimized energy usage extends the network’s lifespan, reducing maintenance needs. Future research could further explore dynamic CH selection using the MFG framework, examine the performance of the protocol with multiple or mobile sinks to enhance adaptability, and focus on real-world experimental validation and integration with other protocols while also addressing security considerations.

## Figures and Tables

**Table 2:** Computational complexity.

Protocol	Complexity
LEACH	O(K)
MFG-LEACH	O(K·T·S)

**Table 3:** Simulation settings [36].

Parameter	Value
Node densities	0.001, 0.01, 0.05 and 0.1 (m^2^)
Initial energy	3 J
Energy step ($\delta_{E}$)	0.03 J
Time	100 s
Time step ($\delta_{t}$)	1 s
Packet size (m)	4000 bits
Number of rounds	15,000
Threshold SINR ($\gamma_{\mathrm{th}}$)	10^−2^
SINR step ($\delta_{\gamma }$)	10^−4^

**Table 4:** Performance Improvement of MFG-LEACH (%).

Node Density	Energy Consumption (%)		
	EAMR	EZ-SEP	BRE
0.001	73.36	73.74	73.88
0.01	50.88	46.80	52.15
0.05	65.0	23.84	16.10
0.1	115.51	57.66	33.97

## Data Availability

Data are contained within the article.

## Appendix A

**Table A1:** Notations.

Variable	Definition
P(CH)	Probability threshold of becoming a CH
*P*	Percentage of nodes designated to become CHs
*r*	Current round number
$E_{k}$	Energy expended by node *k* during transmission
$E_{\mathrm{elec}}$	Node electronics energy
$E_{\mathrm{fs}}$	Energy required for amplification of a signal in open space
$E_{\mathrm{amp}}$	Energy required for amplification of a signal in multi-path model
*m*	Size of packet
*d*	Distance from a transmitter (node or CH)
$d_{\mathrm{th}}$	Predefined threshold distance
γ	Signal-to-interference plus noise ratio (SINR)
$\gamma_{\mathrm{th}}$	SINR threshold
$E_{\min}$	Lower bound on $E_{k}$
$E_{\max}$	Upper bound on $E_{k}$
$E^{r}$	Remaining battery energy
m(t,s)	Mean field
I	Indicator function
